# Code Red in the Supply Center: The Impact of Immune Activation on Hematopoiesis

**DOI:** 10.3390/cells11091586

**Published:** 2022-05-09

**Authors:** Katherine C. MacNamara, Martijn A. Nolte

**Affiliations:** 1 Department of Immunology and Microbial Disease, Albany Medical College, Albany, NY 12208, USA; 2 Department of Molecular Hematology, Sanquin Research, Plesmanlaan 125, 1066 CX Amsterdam, The Netherlands; martijnnolte@gmail.com; 3 Landsteiner Laboratory, Academic Medical Centre, University of Amsterdam, 1105 AZ Amsterdam, The Netherlands

This Special Issue entitled “The Impact of Immune Activation on Hematopoiesis” aims to bring together review and primary articles focused on distinct types of immune activation that impact hematopoiesis. Together, the published papers support an essential and pervasive role of immune activity in shaping the hematopoietic system during development, disease, and aging. Immune activation and states of inflammation occur throughout life, where they drive the emergence of hematopoietic stem cells (HSCs) in the embryo, maintain homeostatic blood production, sculpt specific responses to pathogens, and contribute to hematopoietic dysfunction in disease and aging. The many experiences we have, such as infection, injury, and disease, challenge our hematopoietic system resulting in transient or persistent shifts in blood production. Whether our hematopoietic system recovers to an original “baseline” or re-establishes a new baseline is unknown (Figure 1). Furthermore, immune activity can have both positive and negative outcomes on hematopoietic function, and host survival, that depend on the type of immune activation, the duration of inflammatory signals, and the context, circumstance, or developmental timing of immune activity.

Inflammation modulates fetal hematopoiesis and can increase or reduce production of cell types that emerge during this developmental window, such as B-1 cells and tissue-resident macrophage populations, but less is known about how maternal inflammation may impact definitive HSC emergence. Otsuka et al. used a model of maternal exposure to cytomegalovirus (CMV), the most common congenital infection in humans, and demonstrated the developing cochlea is a sensitive readout of inflammation [1]. The cochlea was populated with immune cells during embryogenesis, a process altered by perinatal CMV infection that correlated with hearing loss. These findings support the idea that development of the hematopoietic system is linked to normal organogenesis and health in the post-natal period. Aluri et al. comprehensively review the requirements for TLR signaling in immune system development and discuss the redundant and non-redundant TLR-dependent signaling pathways that provide host defense against infections [2]. Recent evidence from humans demonstrates a damaging effect of unrestricted TLR8 activation that contributes to a hyper-inflammatory syndrome with increased risk of infection, neutropenia, and bone marrow failure. Therefore, while appropriate inflammatory signals are fundamental to host defense, overactive immune activity can lead to a paradoxical decrease in host defense by promoting hematopoietic dysfunction. 

An elegant feature of the hematopoietic system is its ability to rapidly adapt to environmental signals resulting in expedited production of specific cell types in high demand, often at the expense of other cell lineages. In the context of infection, where control of invading organisms is a matter of life and death, the speed of such responses is likely paramount. How HSC responses are regulated at the molecular level is an area of intense investigation. Chavez et al. address whether functional heterogeneity among HSCs is dependent upon the expression of the transcription factor PU.1, which is known to drive myelopoiesis during infection. Using reporter mice that allow an analysis of PU.1 expression, they demonstrated that IL-1 receptor signaling results in marked changes to the function of HSCs [3]. Low PU.1 expression was previously shown to characterize HSCs with long-term potential; however, chronic IL-1 exposure promoted a functional shift of these cells resulting in increased megakaryocyte potential and increased platelets. Thus, chronic IL-1R-dependent signaling directs myeloid cell responses to inflammatory stress via distinct levels of PU.1 expression. 

The cell source of these inflammatory factors has also been an area of intense research, and T lymphocytes, endothelial cells, and myeloid cells play key roles in driving ‘emergency myelopoiesis’ in different conditions. Goedhart et al. identified a unique population of dendritic cells (DCs) residing in the bone marrow and localized near the vasculature that is exquisitely sensitive to fungal pathogens [4]. DCs in the marrow exhibited efficient phagocytosis, relative to spleen-derived DCs, that depended on increased expression of the c-type lectin Dectin-1. Bone marrow DCs expedited neutrophil differentiation of HSPCs demonstrating a novel cellular mechanism whereby infections are sensed by the hematopoietic system. This rapid dialogue between mature immune cells, cytokines and growth factors and their progenitors highlights the role of adaptation in host defense and suggests that rapid production of granulocytes, including platelets and neutrophils, is fundamental to host defense.

The mammalian immune system must be carefully balanced as both inadequate host defense and over-active immune responses can result in pathology and death. Potent host defense effector mechanisms efficiently kill infected cells but can cause collateral damage if unchecked. Despite a sophisticated immune system, infections are a major killer worldwide and antibiotic therapy is a common intervention in medicine for treating bacterial infections. Based on the observation that long-term antibiotic use in humans is associated with neutropenia, Han et al. investigated mechanisms that drive antibiotic-induced bone marrow suppression. Broad-spectrum antibiotic use in mice resulted in neutropenia and correlated with a profound loss of regulatory T cells in the bone marrow, a site typically enriched for these cells [5]. Whether hematopoietic suppression in antibiotic treatment is a direct result of the loss of regulatory T cell functions, such as IL-10 production, or due to increased inflammation in the bone marrow is unclear. 

Bone marrow suppression can also occur with antifungal and antiviral medications, which is particularly challenging in the clinical scenario of graft-versus-host disease (GvHD), a major complication seen in allogeneic HSC transplantation. A patient’s hematopoietic system must first be ablated and then reconstituted resulting in periods of susceptibility to infection often requiring antibiotics, antivirals, and antifungals. GvHD impacts a variety of tissues in the body, and Muskens et al. describe the particularly insidious effect of GvHD on bone marrow function. T cells play a central role in pathogenesis through the expression of the Fas ligand, which induces apoptosis of both hematopoietic and stromal cells, and through exuberant production of cytokines. However, the precise factors resulting in bone marrow failure may vary from patient to patient and how to preserve hematopoiesis and combat GvHD remains challenging [6]. Similar to GvHD, acquired forms of aplastic anemia arise due to T cell-mediated destruction of the bone marrow, culminating in the loss of all blood lineages. Supporting a central role of T cells in disease pathogenesis is the efficacy of anti-thymocyte globulin (ATG) in treating aplastic anemia, a mainstay therapy consisting of polyclonal antibodies that lead to T cell apoptosis. Tjon et al. compare ATG preparations and clinical outcomes across a variety of multi- and single-lineage marrow failure syndromes and reveal that ATG is most effective against aplastic anemia. Whereas ATG can improve overall survival and hematologic outcomes in aplastic anemia, the benefit of ATG in hypocellular myelodysplastic disease is limited to hematological improvements, with little impact on survival [7]. Though the prevailing paradigm regarding the efficacy of ATG is via T cell destruction, observations suggest that additional immune-modulatory mechanisms may contribute to the benefits of ATG.

Throughout life, we are exposed to pathogens, suffer injuries, and undergo various forms of stress. These experiences drive transient or persistent shifts in blood production and may have long-term impacts on our hematopoietic system. At the same time, the process of aging itself is associated with a general increase in inflammation. Hematological changes associated with aging include increased incidence of myelodysplastic disease (MDS), increased myeloid cancer, reduced HSC function, clonal hematopoiesis of indeterminate potential (CHIP), and loss of lymphoid cell production. Mutations that lead to clonal outgrowth of HSCs or progenitors accumulate during aging and are now well-established to drive MDS. Lynch et al. review key concepts of MDS pathophysiology with a focus on the role of the marrow microenvironment [8]. Targeting aberrant inflammation has emerged as an important therapy for MDS but precisely how hematopoietic and stromal cells interact to drive disease is an area of intense interest with important clinical implications. Bousounis et al. review how aging and inflammation impact the bone marrow niche where HSCs reside [9]. Profound reorganization of HSC location is seen in aging, with simultaneous changes to the composition of stromal cells. Ultimately, these changes culminate in reduced production of important hematopoietic factors and impaired HSC function. Yang et al. review the increasing heterogeneity that exists among HSCs in aged animals and compile and describe key parameters that have been used to identify functional populations of HSCs, which are confounded under inflammatory conditions [10]. Aging of the hematopoietic system is particularly challenging to study due to the complex interactions between HSC and the bone marrow niche, while also considering the interconnected systemic differences that occur during aging such as metabolic, cardiovascular, and skeletal-muscular changes. 

An emerging concept from the articles in this Special Issue suggests transient or temporary immune activation signals are necessary for appropriate hematopoietic responses, whereas persistence of such signals, or failure to resolve inflammation, leads to impaired HSC function resulting in aberrant blood cell generation. A complete understanding of these pathways and defining mechanisms of persisting inflammation may provide novel avenues for treating hematological disorders and/or diseases where aberrant blood production contributes to pathology.

## Figures and Tables

**Figure 1:**
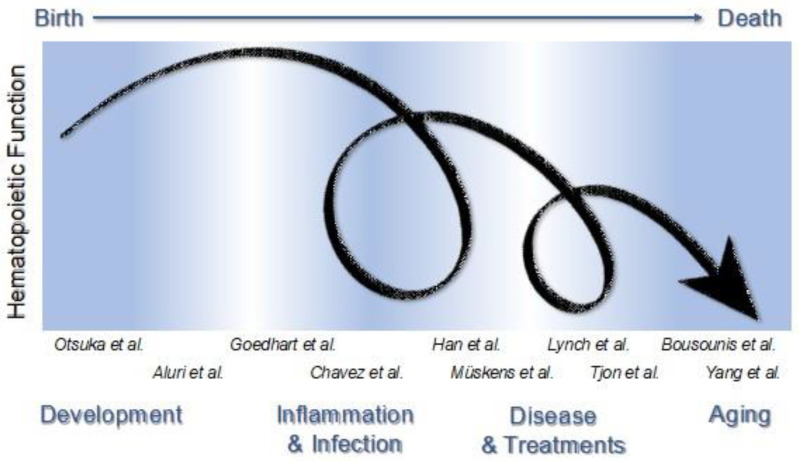
Hematopoietic function changes during life as a result of various challenges. The immune system impacts the very source of blood and immune cells in a variety of settings that span a lifetime. Hematopoietic function can change as a result of immune system activation occurring during normal development and aging, as well as in pathological conditions such as infection and disease. The articles in this Special Issue cover a variety of topics including development [1,2], infection and inflammation [3,4], diseases and their treatments [5,6,7,8], and aging [9,10], and focus on mechanisms whereby the immune system impacts blood cell production.
